# Rational Design of Porous Carbon Hosts for Silicon/Carbon Anodes in Lithium-Ion Batteries: Controlled Synthesis, Silicon Incorporation, Carbon Coating, and Electrochemical Applications

**DOI:** 10.3390/molecules31142483

**Published:** 2026-07-16

**Authors:** Anrui Li, Simin Hua, Yidan Tang, Le Sun, Qinsi Shao, Delun Zhu, Ruicheng Bai

**Affiliations:** 1 School of Materials Science and Engineering, Shanghai University, Shanghai 200072, China; 18663621362@shu.edu.cn (A.L.); huasm@shu.edu.cn (S.H.); tyd@shu.edu.cn (Y.T.); 2 State Key Laboratory of Advanced Refractories, Shanghai University, Shanghai 200444, China; 3 Shaoxing Institute of Technology, Shanghai University, Shaoxing 312000, China; sunle321@126.com; 4 Institute for Sustainable Energy, School of Sciences, Shanghai University, Shanghai 200444, China; qinsishao@shu.edu.cn; 5 Department of Polymer Science and Engineering, Zhejiang University, Hangzhou 310058, China

**Keywords:** porous carbon, Si/C anodes, lithium-ion batteries, pore-structure regulation, silicon incorporation, carbon coating, industrialization

## Abstract

Silicon/carbon (Si/C) composites combine the high theoretical specific capacity of silicon with the electronic conductivity, structural stability, and volume-buffering capability of carbon, making them promising anode candidates for next-generation high-energy-density lithium-ion batteries. However, the substantial volume variation of silicon during repeated charge/discharge processes continuously perturbs the electrode/electrolyte interface, and the resulting interfacial instability remains a major barrier to practical application. Porous carbon host design and Si/C interface regulation have become key routes for improving structural robustness and electrochemical performance. Most existing reviews focus on the failure mechanisms of silicon-based anodes or the structural classification of Si/C composites, whereas the structural regulation role of porous carbon hosts has not been systematically summarized. This review places porous carbon hosts at the center of analysis and summarizes the main preparation strategies, including the hard-templating method, soft-templating method, combined hard- and soft-templating method, template-free synthesis, and etching strategies, with emphasis on their pore-forming mechanisms, structural regulation features, and industrialization potential. Building on this host-centered framework, silicon incorporation and carbon coating strategies are further discussed in terms of their effects on silicon distribution, Si/C interfacial stability, electronic transport, and volume-expansion accommodation. This review further evaluates recent advances in Si/C anodes for lithium-ion batteries from the perspectives of initial Coulombic efficiency, cycling stability, and practical electrode performance. Finally, key challenges related to scalable preparation, structural consistency, electrode-processing compatibility, and industrial adaptation are identified, and future directions for porous-carbon-host-based Si/C anodes are proposed.

## 1. Introduction

Lithium-ion batteries (LIBs) have been widely used in portable electronic devices, electric vehicles, and energy storage systems because of their high energy density and long cycle life [1,2,3]. The continuous demand for higher energy density has pushed conventional graphite anodes close to the performance limit. Graphite delivers a theoretical specific capacity of only 372 mAh/g and lithiates at a potential close to that of Li/Li^+^, leaving limited room for next-generation LIBs [4,5]. Silicon-based anodes have consequently attracted extensive attention as promising alternatives to graphite, although practical application remains limited by intrinsically low electronic conductivity and drastic volume changes during cycling. During repeated charge/discharge processes, the volume change of silicon can exceed 300%, readily inducing electrode pulverization and active-material detachment. Meanwhile, the repeated expansion and contraction of silicon continuously rupture and regenerate the solid electrolyte interphase (SEI), consuming electrolyte and accelerating capacity fading [6,7,8]. Improving silicon utilization while preserving structural integrity and interfacial stability thus remains a central design issue for silicon-based anodes.

Silicon/carbon (Si/C) compositing has become a key strategy in silicon-based anode research, as it provides a practical route to reconcile the high theoretical specific capacity of silicon with the structural stability required for repeated cycling. In Si/C composites, the carbon host buffers silicon volume expansion and establishes a continuous conductive network, while regulated surface chemistry contributes to stabilizing the electrode/electrolyte interface [9]. Among various carbon materials, porous carbon is particularly attractive as a silicon-loading host because of its tunable pore architecture, high specific surface area, and good chemical stability [10,11]. The pore-size distribution, silicon-loading mode, and surface chemistry of porous carbon govern silicon dispersion, ion-transport behavior, and stress transfer within the composite structure, thereby affecting the electrochemical performance of Si/C anodes [12,13,14].

**Figure 1:**
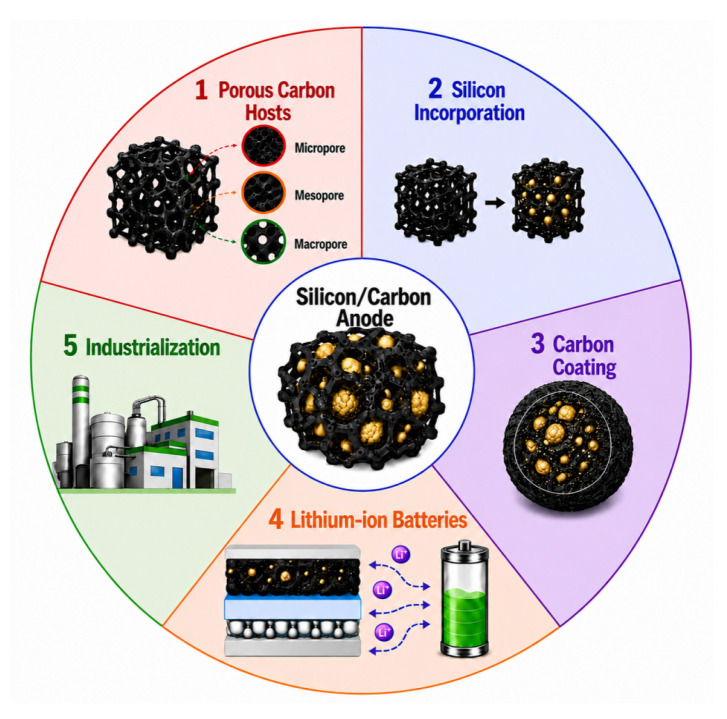
Schematic illustration of the fabrication and application of the silicon/carbon anode for lithium-ion batteries.

In recent years, construction technologies for porous carbon hosts have advanced rapidly. Templating and template-free strategies allow micropores, mesopores, and macropores to be regulated within the same carbon host, providing a more flexible structural basis for confined silicon loading and volume-expansion accommodation [15,16]. Meanwhile, silicon incorporation and surface coating strategies have been continuously refined to improve Si–C interfacial compatibility, reinforce composite structural stability, and enhance cycling stability and rate capability [17]. As shown in Figure 1, this review is organized around the controlled preparation of porous carbon hosts, silicon incorporation strategies, surface coating strategies, lithium-ion battery applications, and industrial perspectives, and discusses their relationships with electrochemical performance. The pore-size distribution, pore volume, specific surface area, and pore-channel connectivity of porous carbon hosts provide the structural basis for confined silicon loading, while also affecting electrolyte infiltration, ion diffusion, and volume-expansion accommodation. On this basis, different silicon incorporation methods further determine the dispersion state, loading amount, and Si–C contact interface of silicon within the carbon framework, thereby influencing the reversible capacity, interfacial stability, and structural retention of the composites. Surface carbon coating subsequently improves electrode interfacial stability, suppresses continuous side reactions between the electrolyte and active silicon, and helps maintain electron-transport pathways and overall structural integrity. Therefore, the coordinated regulation of porous carbon structures, silicon incorporation, and carbon coating plays a decisive role in governing the initial Coulombic efficiency, reversible capacity, rate capability, and long-term cycling stability of Si/C anodes. In addition, the applicable boundaries of different strategies are examined from the perspective of scalable production and industrial adaptation. By establishing a host-centered framework, this review aims to clarify the structure–performance relationships, design principles, and scalable preparation routes for high-performance Si/C anode materials.

## 2. Preparation Methods of Porous Carbon Hosts

Porous carbon hosts govern silicon loading, ion transport, and structural stability in Si/C composite anodes. They also provide spatial support for uniform silicon dispersion, volume-expansion buffering, and electrode interfacial stability. Micropores increase interfacial contact and provide more active sites. Mesopores facilitate electrolyte infiltration and Li^+^ diffusion. Macropores accommodate silicon volume changes during charge/discharge processes and alleviate mass-transfer limitations under high-rate conditions [18,19]. Rational regulation of the proportion, connectivity, and spatial distribution of pores at different scales is essential for improving capacity utilization, rate capability, and cycling stability of Si/C composite anodes.

Various preparation methods have been developed in recent years for the directed construction of porous carbon hosts. According to differences in pore-forming mechanisms and structural regulation modes, these methods mainly include hard templating, soft templating, combined hard- and soft-templating, template-free synthesis, and etching strategies [20,21]. Hard- and soft-templating methods rely on external structure-directing agents to regulate pore size, morphology, and ordering, and are therefore suitable for constructing hierarchical porous structures with relatively controllable pore-size distributions. The combined hard- and soft-templating method integrates the structural advantages of different templates and further regulates the matching relationship among micropores, mesopores, and macropores. Template-free methods mainly generate pores through the intrinsic composition and molecular structure of precursors, together with volatile release, carbon host shrinkage, and structural reconstruction during pyrolysis. Their processes are relatively simplified. Etching strategies further regulate pore quantity, pore-size distribution, and surface structure by selectively removing specific components from the precursor or carbon host. Although some cited examples are not directly based on Si/C anodes, their discussion is still relevant to the design of porous carbon hosts for Si/C systems. This is because the formation and regulation of pores in carbon materials are largely governed by general structural-evolution principles, such as template-directed pore replication, phase separation, gas-release-induced pore generation, selective removal of sacrificial components, and carbon-framework reconstruction during pyrolysis or etching. These mechanisms determine pore size, pore volume, pore connectivity, surface defects, and heteroatom-containing functional sites, which are also key structural parameters for Si/C anodes. For silicon-based anodes, these parameters further influence Si loading uniformity, electrolyte infiltration, Li^+^ transport, electron-transfer pathways, volume-expansion accommodation, and interfacial side reactions. Therefore, porous carbon strategies developed in other battery systems can provide useful guidance for constructing Si/C hosts, especially in terms of hierarchical pore design, interconnected transport channels, and surface-chemistry regulation. Nevertheless, their transfer to Si/C anodes still requires further adaptation according to the specific requirements of silicon loading, expansion buffering, SEI stability, electrode density, and full-cell compatibility.

### 2.1. Hard-Templating Method

The hard-templating method is used to prepare porous carbon materials with well-defined pore sizes. In this method, the precursor of the target material is introduced into the template channels and carbonized through heat treatment. After template removal by calcination or chemical dissolution, porous materials with an inverse structure relative to the original template are obtained [22,23]. Since the template size, morphology, and packing mode affect the pore-size distribution, pore volume, and pore arrangement of the final carbon framework, hard templating is advantageous for constructing structurally controlled carbon hosts.

The main advantage of hard templating lies in its structural precision, but different templates offer different balances between pore controllability and practical feasibility. Ordered templates, such as molecular sieves, mesoporous SiO_2_, and SiO_2_ colloidal crystals, can generate carbon hosts with narrow pore-size distributions and regular pore networks. These features are useful for model studies because the effects of pore size, pore volume, and specific surface area can be separated more clearly. Yang et al. [24] selected ZTC, CMK-3, and OMC-8 as model carbon frameworks with different pore structures and systematically investigated the effects of pore size, pore volume, and specific surface area on CVD silicon deposition and Si/C anode performance. This study shows that silicon deposition during CVD is not simple pore filling, but is governed by precursor transport, adsorption, decomposition, and nucleation inside the pore network. A higher specific surface area increases the contact interface between SiH_4_ and the carbon framework, thereby increasing the probability of intrapore nucleation. Suitable pore volume provides space for silicon deposition and also reserves buffering volume for subsequent lithiation-induced expansion. Pore size further determines the confinement strength of the pore walls and the growth mode of the silicon phase. In microporous hosts, the adsorption potentials from adjacent pore walls tend to overlap, giving stronger adsorption toward the silicon precursor. As a result, silicon preferentially nucleates inside the pores and forms small, uniformly dispersed domains. This structure improves silicon utilization, shortens solid-state Li^+^ diffusion pathways within the silicon phase, and distributes volume variation into numerous confined local spaces, thereby alleviating stress concentration. In contrast, mesoporous hosts with insufficient pore volume and surface area tend to induce further silicon deposition on the external surface after pore filling, forming thick silicon layers that lengthen Li^+^ diffusion pathways and generate stronger stress gradients during cycling. Excessively large mesopores may facilitate electrolyte access and provide more deposition space, but the weakened pore-wall confinement promotes silicon growth, aggregation, and even crystallization, leading to less uniform lithiation and delithiation reactions. Therefore, the design of hard-templated carbon hosts should not simply pursue larger pore size or higher pore volume, but should coordinate specific surface area, pore volume, pore size, and pore connectivity to match silicon deposition, electrolyte accessibility, and Li^+^ transport requirements.

**Figure 2:**
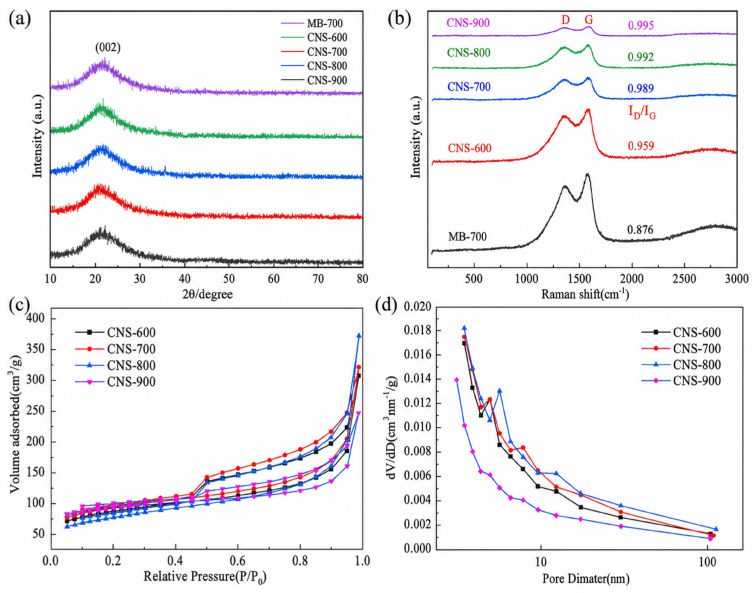
(**a**) XRD patterns of MB-700, CNS-600, CNS-700, CNS-800, and CNS-900. N_2_ adsorption–desorption curves and pore-size distributions of MB-700 and CNS-X (X = 600, 700, 800, and 900). MB-700 denotes the template-free carbon derived from methylene blue at 700 °C. CNS-X denotes N/S co-doped carbon nanosheets prepared using expanded vermiculite as the hard template and methylene blue as the carbon and N/S source, followed by carbonization at X °C; (**b**) Raman spectra of MB-700, CNS-600, CNS-700, CNS-800, and CNS-900; (**c**) the nitrogen adsorption and desorption curves of vermiculite-based template carbon materials calcined at four different temperatures; (**d**) and the pore size distribution curves of vermiculite-based template carbon materials calcined at four different temperatures. Adapted from Ref. [25] under the Creative Commons Attribution 4.0 International License (CC BY 4.0).

The same logic also applies to the selection of template types. Highly ordered templates offer strong pore-structure controllability and are suitable for mechanistic studies or high-performance model Si/C anodes, but their synthesis and removal often require complex procedures and corrosive etchants. Natural mineral templates and low-cost inorganic templates sacrifice part of the structural regularity, but they are more attractive for scalable preparation. Yang et al. [25] used expanded vermiculite as a natural layered hard template and methylene blue as both the carbon source and N/S precursor to prepare N/S co-doped carbon nanosheets. As shown in Figure 2, the N_2_ adsorption–desorption curves show typical type-IV isotherms with H3 hysteresis loops, and the pore-size distribution indicates the formation of mesopore-dominated slit-like pores.

To further improve pore hierarchy, hard templating can be combined with template occupation, foaming, activation, or decomposable-template processes. Yargıç et al. [26] introduced Santa Barbara Amorphous-15 (SBA-15) into a biopitch foaming system and combined it with KOH activation to regulate the pore structure, specific surface area, and pore volume of carbon foams. This strategy can construct mesopore–macropore composite structures and improve pore openness, which is beneficial for electrolyte penetration and long-range mass transport. However, excessive porosity may weaken the mechanical strength of the carbon framework, and incomplete template removal can block internal pores. Zhu et al. [27] used nano-CaCO_3_ as a decomposable hard template and lignin as the carbon source to prepare hierarchical porous carbon. During carbonization, nano-CaCO_3_ acts as a pore-forming template, while CO_2_ released from its decomposition further activates the carbon framework and expands part of the micropores and small mesopores. Compared with conventional inert templates, this strategy couples template decomposition with in situ activation, which is useful for constructing mesopore-rich hierarchical networks. These examples indicate that effective ion transport depends not only on BET surface area and total pore volume, but also on whether the pores are open, connected, and accessible to the electrolyte.

Template cost and removal complexity are critical for practical Si/C anode production. Highly ordered templates usually provide better structural precision, but their preparation and removal increase processing cost and environmental burden. Nahda et al. [28] used silica gel 60 instead of highly ordered templates such as SBA-15 and SBA-16. Although this low-cost template shows lower pore regularity and replication accuracy, it offers broader raw-material availability and better process adaptability. Therefore, the suitable template for Si/C anodes should be selected according to the target application. Ordered templates are more suitable for mechanism-oriented studies and precise pore engineering. Natural mineral or low-cost silica templates are more promising for scalable synthesis. Decomposable templates and activation-assisted templates are useful when hierarchical pore networks and improved electrolyte accessibility are required.

Hard templating allows template size, particle stacking, and space-occupation modes to be regulated more clearly. Carbon hosts with well-defined pore characteristics can then be produced. However, its limitations are also clear. The impregnation and template-removal processes are relatively complex. Acid/alkali treatment may also cause environmental pollution and structural damage [29].

### 2.2. Soft-Templating Method

Soft templating is mainly based on the self-assembly of surfactants, natural polymers, or block copolymers to direct pore formation in carbon frameworks [30]. Unlike hard templating, which relies on rigid-space replication, soft templating depends on molecular interactions between templates and carbon precursors. Hydrogen bonding, electrostatic interactions, coordination interactions, and hydrophilic–hydrophobic microphase separation can regulate precursor distribution and template aggregation. During carbonization, the organic template decomposes or is released, leaving pore structures related to the original assembled domains. Soft templating usually avoids harsh template-removal procedures and provides a flexible route for constructing mesopore-dominated carbon hosts [31]. Its structural precision is generally lower than that of hard templating, and the resulting pore structure is more sensitive to precursor chemistry, solvent environment, heating process, and template–precursor compatibility [32].

The interfacial-regulation ability of natural polymer soft templates is particularly relevant to Si/C anodes. Chitosan (CTS) contains amino and hydroxyl groups that can interact with silicate species through electrostatic attraction and hydrogen bonding. These interactions help regulate reactant distribution on the silicon surface and promote uniform reconstruction of the oxidized interface. Wang et al. [33] used photovoltaic silicon cutting waste as the silicon source and employed CTS to assist in regulating the silicon surface oxide layer, forming a SiO_x_-containing interfacial structure and dual carbon coating. In this case, CTS participates in surface oxide reconstruction, carbon-interface regulation, and structural construction. Such a strategy is suitable for low-cost silicon sources or recycled silicon wastes, where surface defect control, interfacial stress relief, and stable Li^+^ migration across the SiO_x_/C interface are closely related to silicon utilization and cycling stability.

By comparison, surfactant-assisted soft templating mainly improves carbon host morphology and electrolyte accessibility. CTAB-type small-molecule surfactants can regulate spherical assembly, internal cavities, and local hierarchical pores through electrostatic interactions and coordination-assisted crosslinking. Liang et al. [34] used cetyltrimethylammonium bromide (CTAB) and alkali lignin to construct hollow hierarchical porous carbon spheres, showing that small-molecule surfactants are effective for morphology control and cavity construction. Such hollow or hierarchical structures can provide larger electrolyte-contact areas and shorter ion-diffusion pathways. However, compared with block-copolymer templates, small-molecule surfactants usually have weaker ability to construct long-range ordered mesoporous networks. Their advantage lies in flexible morphology regulation, while their limitation lies in less predictable pore ordering and possible structural shrinkage during carbonization.

Block copolymer templates provide better mesopore regularity because their hydrophilic and hydrophobic segments can self-assemble into ordered domains. P123 and F127 are typical Pluronic-type templates, but their design roles are different. P123 is often more effective for directing mesopore formation and regulating carbon host connectivity. Shi et al. [35] used P123 with sodium oleate to prepare nitrogen-doped hollow carbon nanospheres, indicating that P123 can contribute to mesopore formation, interfacial stabilization, and nitrogen-doping regulation. When P123 is introduced into a biopitch foaming system, as reported by Yargıç et al. [36] its role extends from molecular assembly to larger-scale foam-cell regulation and pore-connectivity optimization. F127 relies more on hydrogen bonding with oxygen-, nitrogen-, or sulfur-containing precursors and is suitable for surface-functionalized porous carbons. Zhang et al. [37] used F127 with tannic acid to prepare N/S co-doped porous carbon, showing that F127-based assembly can combine pore formation with defect and surface-chemistry regulation. Therefore, P123 is more suitable for pore-channel and framework regulation, whereas F127 is more useful for molecular assembly and heteroatom-rich surface construction.

The processing route is another important criterion for evaluating soft templating. Conventional soft-template assembly often requires solvent-mediated micelle formation, which improves molecular-level mixing but may introduce solvent consumption, drying shrinkage, and batch-to-batch variability. Li et al. [38] proposed a solvent-free soft-templating strategy in which amino acid additives regulated micelle configuration and pore-size structure. This strategy shifts soft-template design from simple template selection toward control of micelle-packing parameters and molecular interactions. It also reduces solvent use and wastewater-treatment pressure, which is important for scalable preparation. Nevertheless, solvent-free or low-solvent systems still require precise control of precursor mobility, template dispersion, and carbonization-induced shrinkage to maintain pore uniformity.

Soft templating is suitable for constructing mesopore-dominated carbon hosts with tunable surface chemistry. Its main advantages are mild assembly, simplified template removal, flexible surface functionalization, and good compatibility with heteroatom doping or interfacial modification. Its limitations include relatively weak macropore construction, lower pore-size precision than hard templating, and sensitivity to precursor–template interactions. For Si/C anodes, soft templating is most useful when the design goal is to improve electrolyte accessibility, stabilize Si/C or SiO_x_/C interfaces, introduce functional heteroatoms, and construct mesoporous ion-transport channels. Future soft-templating strategies should further combine predictable micelle assembly, low-solvent or solvent-free processing, and hierarchical pore construction to improve silicon utilization, Li^+^ transport, interfacial stability, and practical scalability.

### 2.3. Combined Hard- and Soft-Templating Method

The combined hard- and soft-templating method is developed to overcome the structural limitations of single-template routes [39]. Hard templates provide more reliable control over external morphology, cavity size, and spatial layout, while soft templates are more effective in directing mesopore formation inside carbon walls and regulating local surface chemistry. Their combination allows pore formation to be regulated at different length scales within one carbon host. This is useful for Si/C anodes because silicon loading, electrolyte penetration, Li^+^ transport, and volume buffering usually require different pore domains to work together rather than a single type of pore [40,41].

Compared with single hard templating, the combined route can reduce the formation of dense carbon walls and improve internal mesoporosity. Compared with single soft templating, it can better retain large cavities, defined host morphology, and long-range structural integrity. Therefore, the key value of this strategy lies in coordinating host morphology, carbon-wall mesoporosity, pore connectivity, and surface chemistry. The hard template mainly determines the overall spatial framework and reserved voids, while the soft template adjusts mesoporous channels and local chemical environments. This division of structural roles can improve the compatibility between silicon accommodation and ion transport.

Different studies show how this coordination can be realized through different template combinations. Zhong et al. [42] combined wrinkled mesoporous SiO_2_ nanospheres with F127 micelles to construct hierarchical porous carbon hosts. In this system, the SiO_2_ template controlled the host spacing, large-scale pore layout, and carbon-wall thickness, while F127 contributed to mesopore formation within the carbon framework. This design is suitable when continuous mesoporous channels need to be introduced into a defined host morphology. Hu et al. [43] used Fe_2_O_3_ microcubes together with F127 and 1,3,5-trimethylbenzene to form carbon structures with internal cavities and mesoporous shells. Compared with the former system, this design gives a clearer functional division between the internal cavity and the transport shell, making it more suitable for structures requiring reserved expansion space and ion-accessible carbon walls. Chen et al. [44] further combined reaction-generated boiling bubbles with dual-sized SiO_2_ hard templates. This method broadened the pore-size distribution by using transient soft templates and solid hard templates simultaneously, which is advantageous for constructing more complex hierarchical networks.

The combined hard- and soft-templating strategy is suitable for constructing carbon hosts with coupled pore hierarchy and wall-level mesoporosity. Its advantage lies in integrating the shape-retention capability of hard templates with the mesostructure-directing ability of soft templates, thereby achieving a more balanced host architecture than single-template routes. However, this structural flexibility also increases synthetic complexity. Mismatched template size, surface chemistry, assembly sequence, or removal conditions may lead to incomplete mesopore formation, fragile carbon walls, blocked pore networks, or poor batch reproducibility. Multi-template systems also increase precursor-formulation complexity and template-removal difficulty, which limits their direct scalability. Therefore, combined templating is more suitable for mechanism-oriented host design and high-performance Si/C structures requiring precise pore hierarchy. Future development should focus on simplifying template removal, improving template compatibility, and maintaining pore hierarchy, mechanical strength, silicon-loading efficiency, and electrochemical stability in scalable processes.

### 2.4. Template-Free Method

Template-free methods construct porous carbon hosts by relying on the intrinsic composition, spatial organization, and pyrolysis behavior of precursors [45]. Compared with template-assisted methods, they avoid additional template synthesis and removal, which shortens the preparation route and improves raw-material flexibility. This feature makes them attractive for low-cost Si/C anode preparation, especially when carbon host formation, pore generation, heteroatom incorporation, and silicon compositing can be integrated into one process. However, because pore formation is mainly governed by precursor chemistry and thermal decomposition behavior, the pore-size distribution and batch reproducibility are usually less controllable than those of template-directed routes [46,47,48,49].

Early template-free Si/C designs often used volatile species generated during pyrolysis to create pores. Wang et al. [50] used zinc citrate as the precursor, where Zn species were reduced and volatilized at high temperature to generate mesoporous carbon hosts and support subsequent Si/C composite formation. This strategy couples metal transformation, pore formation, and carbon host construction, giving a relatively simple route to mesopore-rich Si/C composites. Its advantage lies in the direct generation of ion-transport channels during carbonization, but its pore structure strongly depends on metal volatilization behavior. Residual metal species, excessive carbon shrinkage, or uneven Zn release may also complicate structural control.

Compared with volatile-metal release, decomposition-induced gas release provides a more chemistry-oriented way to refine the pore structure and introduce functional sites. Miao et al. [51] used p-phenylenediamine sulfate as the precursor. Gas released from [HSO_4_]^−^ decomposition generated ultramicropores and micropores, while N/S sites were introduced into the carbon framework. This design improves local surface chemistry and can promote interfacial reactions or ion adsorption. However, fine pores alone provide limited space for silicon accommodation and volume buffering. Therefore, this strategy is more effective when combined with mesoporous or macroporous frameworks, rather than being used as the only pore-forming mechanism for high-silicon-loading hosts.

Biomass-derived precursors provide a further shift from chemical pore generation to structural inheritance. Xu et al. [52] used sugarcane bagasse as the raw material, preserving the vascular-bundle channels by pre-carbonization and then introducing micropores and mesopores into the tube walls through KOH activation. Compared with gas-release routes, this strategy offers more continuous macroporous channels and better long-range mass transport. Its limitation is that the inherited structure is determined by the natural precursor, so pore precision and structural uniformity remain difficult to control.

**Figure 3:**
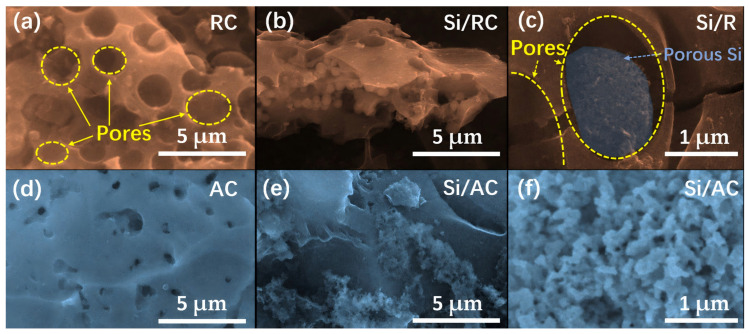
SEM images of RC (**a**), Si/RC (**b**,**c**), AC (**d**) and Si/AC (**e**,**f**). Reproduced from Ref. [53] under the terms of the Creative Commons Attribution 4.0 International License (CC BY 4.0).

Meng et al. [53] further showed that the difference between biomass hosts can directly affect silicon distribution and electrochemical performance. Reed catkin-derived carbon (RC) and apricot shell-derived carbon (AC) were used as low-cost biomass hosts to prepare silicon/reed catkin-derived carbon (Si/RC) and silicon/apricot shell-derived carbon (Si/AC) through carbonization, KOH activation, TEOS hydrolysis, and magnesiothermic reduction. Compared with AC, RC contains richer macropores and mesoporous channels (Figure 3), which helps retain silicon inside the carbon matrix and improves volume buffering and Li^+^ transport. AC has fewer and smaller macropores, so silicon is more likely to accumulate on the external surface, leading to weaker cycling stability. This comparison indicates that biomass selection should consider natural pore hierarchy and silicon-anchoring ability, not only cost and availability. The drawback is that Mg thermal reduction still requires acid treatment to remove by-products and residual SiO_2_, which increases processing complexity.

To reduce the dependence on natural precursor morphology, in situ reaction-network transformation offers a more designable route. Dong et al. [47] induced condensation between a silane coupling agent and phytic acid on a pre-carbonized polyvinylpyrrolidone (PVP)–melamine substrate, followed by carbonization to form a SiO_x_/C composite. Compared with direct biomass inheritance, this strategy allows the carbon network and pore channels to be generated from a designed precursor reaction. It can provide better framework continuity, conductive connection, and structural retention during cycling. Its limitation lies in the higher sensitivity to precursor condensation, crosslinking uniformity, and carbonization shrinkage.

Template-free methods are more attractive when low cost, simplified processing, and precursor versatility are prioritized. Volatile-metal release is useful for mesopore generation and Si/C compositing in one thermal process, while gas-release chemistry improves fine-pore formation and surface functionality. Biomass inheritance provides low-cost macroporous frameworks, and in situ reaction-network transformation offers higher chemical designability and better structural continuity. These methods have shorter processing routes and allow more flexible raw-material selection. They can also be more easily combined with carbon host formation, heteroatom incorporation, and silicon phase compositing. However, this endogenous pore-forming mode is highly sensitive to precursor composition, pyrolysis atmosphere, and heating process. Its pore-structure precision and batch stability therefore remain weaker than those of template-directed routes.

### 2.5. Etching Method

Etching methods introduce reactive media such as acids, alkalis, salts, or reactive gases during heat treatment or post-treatment to selectively consume unstable components in the precursor or carbon host [54]. This process induces pore generation, pore expansion, and local carbon-framework reconstruction. Etching is different from endogenous pyrolysis-driven pore formation because the pore evolution is actively guided by external reactive media [55]. It also differs from template-assisted routes in that the pore structure can be further adjusted after the carbon framework has been formed [56]. Therefore, etching is particularly useful when the initial carbon host lacks sufficient mesopores, accessible channels, or buffering space for silicon incorporation.

KOH activation provides a direct way to expand pore structures and tune pore topology. Deng et al. [57] used petroleum pitch as the carbon source to prepare porous carbon through KOH activation, followed by silicon introduction via chemical vapor deposition (CVD). Their study clarified how pore topology affected silicon deposition and electrode stability. With an increasing KOH dosage, the carbon host evolved from a micropore-dominated structure to a network containing more mesopores and macropores. This gradual pore expansion can increase silicon-storage space and improve electrolyte accessibility. However, excessive KOH etching may over-expand pores, damage pore walls, and weaken the mechanical integrity of the carbon host. Enlarged pores may also reduce silicon confinement and promote nonuniform silicon deposition. Therefore, the key issue in KOH etching is to control the activation degree rather than simply maximize pore volume.

A lower-cost extension is to replace purified chemical etchants with reactive solid wastes. Zhang et al. [58] employed slag as a high-temperature reactive medium rich in alkali-metal and alkaline-earth-metal components. This strategy uses the intrinsic reactivity of industrial waste to activate carbon, which is attractive for cost reduction and resource utilization. It may simplify reagent selection and improve economic feasibility. Its limitation lies in the lower compositional controllability of slag, since its chemical composition varies with source and processing history. Inhomogeneous reaction intensity and residual inorganic species may also make pore regulation less predictable.

When the carbon host already has a designed framework, gas-mediated selective etching can provide local pore refinement while retaining the original architecture. Chi et al. [59] used fluorine-containing gases generated from polytetrafluoroethylene (PTFE) decomposition to remove SiO_2_ templates. This gas-phase process can avoid severe structural collapse caused by strong liquid acid or alkali treatment and can generate additional pores with suitable sizes. It is useful for systems that require template removal, pore opening, and framework preservation at the same time. However, fluorine-containing gases require strict control. Excessive etching may damage the carbon framework, and the use of fluorinated species also increases safety and environmental concerns.

For carbon hosts with poorly developed pores, molten-salt etching provides another route to enhance mesoporosity. Zhou et al. [60] used ZnCl_2_ molten-salt etching to transform a dense precursor into carbon with clear mesoporous characteristics. Under molten-salt conditions, ZnCl_2_ can promote dehydration, aromatization, and mesopore formation, making it useful for surface mesopore enhancement and electrolyte-accessible channel construction. The drawback is that thorough washing is required after etching, and residual chloride or zinc species may affect electrochemical stability. Similar to other etching routes, excessive reaction can also introduce abundant defects and reduce carbon-framework strength.

Etching methods can rapidly regulate the pore structure of porous carbon by enlarging pore size, increasing pore volume, and improving pore connectivity, providing more space for silicon deposition and volume-expansion buffering. Excessive etching, however, may damage the continuous carbon host, introduce excessive defects, and weaken electronic conductivity and mechanical stability. Etching strategies should balance pore-structure regulation with structural integrity.

### 2.6. Summary

The preparation of porous carbon hosts provides the structural basis for Si/C anodes and influences both silicon incorporation and final battery performance. Table 1 compares these methods and provides reference guidance for subsequent research. In terms of pore-structure controllability, hard templating shows the highest precision, hard/soft templating is more suitable for hierarchical pore regulation, soft templating mainly controls mesopores and surface chemistry, etching allows secondary adjustment of pore size and connectivity, and template-free methods show relatively weak precision because their pore structures depend strongly on precursor composition and pyrolysis behavior. Regarding template-removal complexity, template-free methods are the simplest, soft templating usually avoids harsh removal processes, while hard templating and hard/soft templating require more complicated template elimination, and etching may involve corrosive media or repeated washing. From the cost perspective, template-free routes and low-cost etching strategies are more favorable for scalable preparation, whereas ordered hard templates and multi-template systems increase material and processing costs. For silicon-loading efficiency, hard-templated and hard/soft-templated hosts with well-defined pore structures can better match silicon deposition and confinement, etching can increase pore volume and loading space, soft-templated hosts mainly improve electrolyte-accessible mesopores and interfacial regulation, while template-free hosts depend on whether the inherited or reaction-derived pores are suitable for silicon accommodation. The final electrochemical performance is therefore determined by the balance among silicon loading, ion transport, volume buffering, and carbon-framework integrity. The design of porous carbon hosts should first reserve sufficient pore volume and expansion space according to the target silicon content. Micropores or small mesopores should be used to enhance silicon-precursor adsorption and silicon confinement, mesopores should ensure electrolyte infiltration and Li^+^ transport, and an appropriate amount of macropores should relieve electrode-level stress. Excessive pore formation should be avoided because it may reduce tap density, framework strength, and electronic conductivity.

## 3. Silicon Incorporation and Carbon Coating Strategies

The performance improvement of Si/C composite anodes is strongly affected by silicon dispersion in the carbon host, the bonding state, and structural stability. The silicon incorporation method determines the distribution of silicon within the carbon host. Carbon coating mainly reduces direct contact between silicon and the electrolyte, maintains continuous electron transport pathways, and provides mechanical confinement during silicon expansion. As a result, interfacial side reactions and expansion-induced structural degradation can be mitigated. These two strategies substantially affect Si/C anodes through different functions [61].

### 3.1. Silicon Incorporation Strategies

Increasing silicon content is only the first step in silicon incorporation. Silicon incorporation requires effective introduction into the carbon host, uniform nanoscale dispersion, and stable integration with the carbon phase. Researchers have developed several representative strategies, such as chemical vapor deposition (CVD), in situ generation, and mechanical compositing [60,61,62,63,64,65]. Several key aspects of Si/C anode preparation are affected by the incorporation route, including silicon dispersion, interfacial continuity, silicon accommodation capability of the carbon host, and process feasibility.

#### 3.1.1. CVD Method

Chemical vapor deposition (CVD) uses gaseous precursors that decompose or react under heating conditions and deposit reaction products on the substrate surface. Gaseous silicon sources can enter the surface, interlayer regions, or internal pores of carbon materials and generate silicon during thermal decomposition. The value of CVD lies in its ability to regulate silicon deposition location, particle size, and interfacial contact more precisely than mechanical mixing or conventional impregnation methods. High silicon loading cannot be achieved only by increasing precursor supply. If the carbon host cannot adsorb, transport, and confine the gaseous silicon species effectively, silicon may accumulate on the external surface, leading to nonuniform lithiation, higher interfacial side reactions, and poorer cycling stability [63,64,65].

Therefore, the carbon host should be regarded as an active participant in CVD-based silicon incorporation. Its pore volume determines the available space for silicon deposition, pore size controls confinement strength, and pore connectivity affects gas diffusion and precursor utilization. Mo et al. [66] used porous carbon as the host in a fluidized-bed system and introduced a SiH_4_/N_2_ mixed gas. After SiH_4_ entered the pore network, nanosilicon was generated through thermal decomposition within the confined space. The significant decrease in specific surface area and the change in adsorption–desorption behavior after silicon deposition indicate that nanosilicon occupied a large fraction of the original pore space. This result confirms that porous carbon hosts can provide three-dimensional deposition space for highly dispersed silicon loading. However, it also suggests that the pore structure must be carefully matched with silicon deposition, because excessive pore filling may block transport channels and reduce electrolyte accessibility.

**Figure 4:**
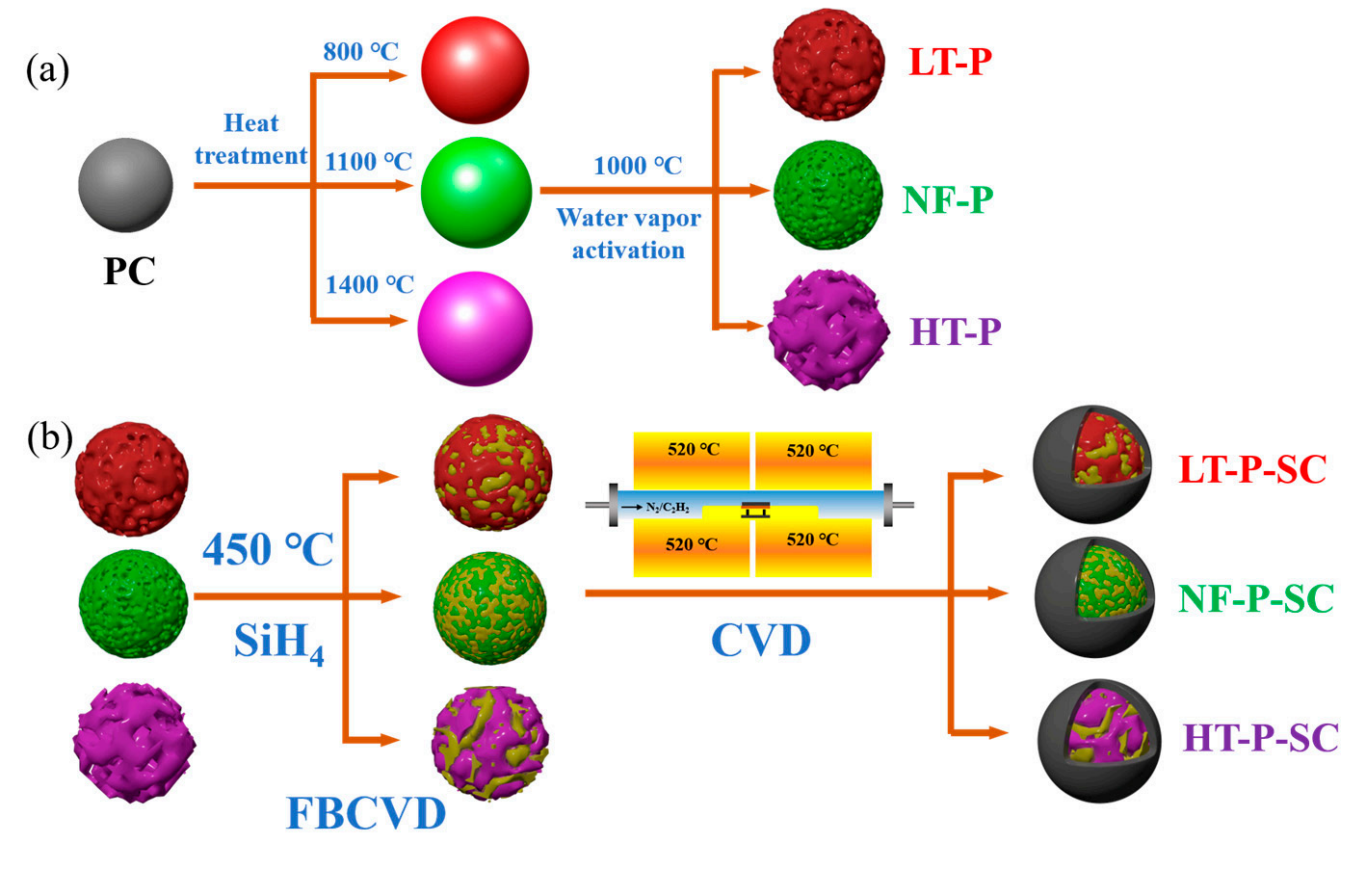
(**a**) Preparation schematic of porous C; (**b**) preparation schematic of Si-C composite [67].

Mei et al. [67] further clarified this structure–deposition relationship by changing the pore-size distribution of biomass-derived hard carbon before SiH_4_ fluidized-bed CVD. The porous carbons were obtained by heat treatment at different temperatures followed by water-vapor activation, and silicon was then introduced by SiH_4_ CVD, as shown in Figure 4. Their results show that micropore-dominated hosts are more favorable for adsorption and confined deposition of SiH_4_-derived silicon, whereas hosts containing excessive mesopores and macropores tend to promote surface silicon deposition and partial silicon crystallization. This distinction directly affects electrochemical performance. The medium-temperature-derived porous carbon achieved a better balance between micropore confinement, mesopore transport, and electronic conductivity, giving the corresponding NT-P-SC electrode a high initial Coulombic efficiency of 94.47% and superior full-cell cycling stability after blending with graphite. This work indicates that the optimal CVD host is not the one with the largest pores or the highest pore volume, but the one that balances silicon confinement, gas diffusion, electron transport, and residual pore accessibility.

CVD is suitable for constructing Si/C anodes that require controlled silicon distribution, nanoscale silicon formation, and intimate Si–C contact. Its performance advantage becomes more evident when porous carbon hosts provide suitable micropores for silicon confinement, mesopores for gas and ion transport, and sufficient conductivity for charge transfer. Nevertheless, CVD involves higher equipment costs and a narrower processing window. In continuous preparation, issues related to powder flow, gas–solid contact, and batch uniformity still need to be addressed.

#### 3.1.2. In Situ Generation Method

The core of the in situ generation method lies in embedding silicon phase formation into the construction of the composite structure. This method does not require prefabricated nanosilicon particles. Instead, the silicon phase is directly transformed from precursors during the reaction and composited with the carbon phase during phase formation. The size, distribution state, and interfacial bonding of silicon can be regulated during the generation process [68]. Compared with externally added nanosilicon, the in situ generation route helps alleviate silicon particle aggregation, uneven dispersion, and interfacial detachment. The continuity of the composite structure and interfacial stability can also be improved.

In Si/C systems, a representative in situ generation route uses inorganic silicon sources such as SiO_2_ as precursors. Si is directly generated through reduction reactions, followed by the formation of a carbon coating layer during subsequent carbonization. Gao et al. [64] used rice-husk-derived SiO_2_ as the silicon source and achieved the in situ reduction in SiO_2_ through a NaH-AlCl_3_ molten-salt system. A carbon coating layer was then constructed from paraffin-derived carbon, producing a Si/C composite structure. This process integrates silicon phase generation with carbon-layer construction in a continuous reaction process. It helps reduce the aggregation risk associated with externally added nanosilicon and improves Si/C interfacial contact.

The in situ generation method reduces cost while forming a favorable interface. It also reduces the dependence on prefabricated nanosilicon and simplifies the overall preparation process. A carbon layer can also form during the reaction, which helps stabilize the Si/C interface.

#### 3.1.3. Mechanical Compositing Method

Mechanical compositing uses ball milling, grinding, or other mechanical mixing methods to bring silicon and carbon sources into close contact under external force. Its main value lies in process simplicity, broad raw-material adaptability, and scale-up feasibility [69]. Ball milling can reduce the distance between silicon and carbon particles, increase their contact area, and provide a relatively uniform starting system for subsequent carbonization, coating, or conductive-network construction. However, mechanical compositing mainly produces physical contact. Without further interfacial strengthening, the Si/C interface is usually unstable, and the silicon distribution is highly dependent on milling intensity, milling time, and particle-size matching [70].

A basic strategy is to use mechanical mixing to disperse silicon first and then fix the structure through pyrolytic carbonization. Han et al. [71] prepared N-doped carbon-embedded Si@C composites by ball milling Si nanoparticles with polyvinylpyrrolidone (PVP), followed by pyrolysis. In this process, ball milling improved the initial dispersion of Si, while carbonization converted PVP into an N-doped carbon host and embedded the pre-dispersed Si particles. This route is simple and suitable for low-cost preparation, and it can form a relatively uniform Si/C distribution at the particle scale. Its limitation is that the interfacial interaction mainly comes from carbonization-induced embedding. The bonding strength between Si and carbon remains limited, and excessive milling may also cause particle aggregation, structural damage, or uneven local stress.

To overcome the weak interfacial coupling of simple mechanical mixing, mechanical compositing can be used as a precursor-organization step before further chemical reconstruction. Hao et al. [70] first mixed microsilicon, graphite, and bamboo charcoal by ball milling to construct a uniform precursor system. During subsequent heat treatment with melamine and cobalt salt, nitrogen-doped carbon nanotubes (N-CNTs) were grown in situ inside and on the surface of the composite, while Si–C covalent bonds were formed. In this case, ball milling mainly ensured initial contact among the components, and the following in situ growth process further built conductive pathways and strengthened the Si/C interface. This design improves electron transport and structural stability more effectively than mechanical mixing alone, especially for microsilicon-based composites that require stronger buffering and interface fixation.

Mechanical compositing is suitable for scalable Si/C preparation when low cost and simple processing are prioritized. Its performance advantage depends on whether the mechanically mixed precursor can be transformed into a stable composite structure in subsequent steps. Direct ball milling followed by carbonization can improve dispersion and reduce preparation complexity, but its interface stability and silicon confinement remain limited. Combining mechanical compositing with in situ carbon growth, heteroatom doping, or covalent interfacial construction can improve conductive connectivity and cycling stability, but it also increases process complexity and requires better control of milling conditions, heat treatment, and catalyst residues. Therefore, mechanical compositing should be regarded as a practical precursor-organization strategy rather than a complete interface-design method by itself.

#### 3.1.4. Summary

The applicability of these three methods should be judged by specific capacity targets, silicon phase distribution requirements, interfacial-stability demands, and engineering conditions. Table 2 compares representative silicon incorporation strategies in terms of their main advantages, limitations, and corresponding structural targets.

### 3.2. Carbon Coating Strategies

In Si/C anodes, carbon coating is introduced after silicon incorporation to reduce direct contact between silicon and the electrolyte, maintain electron transport pathways, and restrain structural deformation during cycling. Silicon formed by CVD or in situ reactions may still be exposed at pore edges, particle surfaces, or imperfect Si/C interfaces. During repeated lithiation and delithiation, these exposed regions can trigger continuous SEI rupture, side reactions, interfacial polarization, and electrode thickening. Therefore, an effective carbon coating should do more than cover the silicon surface. It should match the silicon distribution and carbon host structure, while providing interfacial protection, conductive continuity, and mechanical confinement [72,73].

Lv et al. [74] demonstrated this principle using a porous hard carbon@Si@soft carbon (PHC@Si@SC) structure. After nanosilicon was loaded onto porous hard carbon through CVD, pitch-derived soft carbon was introduced as an outer protective layer. In the uncoated PHC@Si structure, porous hard carbon provides deposition space for silicon, but the contact between the host and the silicon phase is still mainly physical. During cycling, silicon expansion may weaken this contact, interrupt Li^+^ diffusion pathways, and increase charge-transfer polarization. After soft-carbon coating, GITT results suggested faster Li^+^ diffusion, and EIS analysis showed lower charge-transfer resistance and a smaller Warburg factor. These results suggest that the soft carbon layer may improve interfacial ion migration and electronic connection. Post-cycling SEM images further showed that PHC@Si@SC maintained a more intact electrode morphology and exhibited much less thickness expansion than PHC@Si. This comparison suggests that pitch-derived soft carbon may function as a flexible confinement layer and an interfacial bridge. It may compensate for the insufficient stability of the PHC/Si physical interface and contribute to transforming pore-supported silicon deposition into a more stable electrode structure.

Wang et al. [75] further extended carbon coating from a particle-level protective shell to an electrode-level conductive confinement network. In this work, nanosilicon was first deposited inside three-dimensional porous carbon through SiH_4_ CVD, and a reduced graphene oxide (rGO) coating network was then constructed outside the Si/C particles through graphene oxide (GO) hydrothermal self-assembly and thermal reduction. This design differs from pitch-derived soft carbon coating because rGO is expected to provide flexible interparticle connection and external spatial confinement. Cross-sectional SEM results showed that the Si/C/rGO electrode underwent only slight active-layer thickening after 300 cycles, while the uncoated Si/C electrode showed much larger expansion. The retained porous morphology before cycling and partially filled pores after cycling suggest that the internal voids may serve as reserved expansion space, allowing part of the silicon strain to be accommodated within the electrode structure. XPS analysis also showed stronger LiF-related signals in the cycled Si/C/rGO electrode, suggesting the possible formation of a more stable LiF-rich SEI derived from fluoroethylene carbonate (FEC) and LiPF_6_ decomposition. Together with lower charge-transfer resistance, higher Li^+^ diffusion coefficients, and a larger pseudocapacitive contribution, these results suggest that rGO coating may contribute to improved cycling stability through conductive-network construction, particle fixation, SEI stabilization, and faster interfacial charge transfer.

These two studies show that carbon coating should be selected according to the exposed silicon location and the weakness of the original Si/C structure. Pitch-derived soft carbon is more suitable when the main problem is insufficient interfacial contact between silicon and the porous carbon host, because it can form a continuous encapsulation layer and relieve local stress. rGO is more suitable when particle-level connection, electrode-level confinement, and flexible conductive pathways are required. In both cases, the coating layer must balance protection and transport. Insufficient coating cannot suppress electrolyte attack or interface separation, while excessive coating reduces silicon content, increases Li^+^ transport resistance, and may lower the practical capacity. Therefore, carbon coating design should coordinate coating thickness, carbon type, silicon exposure degree, pore accessibility, and electrode-level structural stability.

## 4. Applications of Si/C Anodes in Lithium-Ion Batteries

Si/C anodes can be prepared through carbon host construction, silicon incorporation, and carbon coating. For lithium-ion battery applications, Si/C anodes should be evaluated not only by half-cell capacity, but also by full-cell performance and practical parameters such as initial Coulombic efficiency (ICE), electrode thickness expansion, rate capability, electrode density, and volumetric capacity [76]. From a host-centered perspective, these performance metrics are closely related to the pore architecture of the carbon host. Pore size affects silicon deposition and confinement, pore volume determines silicon-loading space and expansion-buffering capacity, pore connectivity and tortuosity control electrolyte infiltration and Li^+^ transport, and specific surface area influences both precursor adsorption and SEI formation. Therefore, the role of porous carbon hosts should be assessed through their structural contribution to silicon utilization, interfacial stability, transport kinetics, and electrode-level dimensional stability.

The porous carbon host first needs to confine silicon while maintaining sufficient transport pathways. Song et al. [77] used this route to prepare nitrogen-doped porous carbon (NPC)–Si–C composites. In this system, the NPC host provides pore volume for silicon accommodation and expansion buffering, while the connected pore network can shorten Li^+^ transport pathways and reduce local tortuosity. Nitrogen doping may further improve interfacial wettability and electron transport. These host features help connect silicon utilization with ICE, rate capability, and cycling stability. With host confinement and outer carbon coating, the NPC–Si–C anode delivered an initial discharge specific capacity of 1725.17 mAh/g and an initial Coulombic efficiency of 91.02%. After 100 cycles at 0.5 C, the capacity retention reached 75.66%. When paired with a high-Ni LiNi_0.8_Co_0.1_Mn_0.1_O_2_ (NCM811) cathode, the full cell retained 62.25% of its capacity after 150 cycles at 1 C. This result indicates that the regulation of silicon deposition behavior by porous carbon hosts can be further extended to complete cell systems. However, abundant pores and high surface area may also increase electrolyte contact and irreversible SEI formation, so outer carbon coating and suitable surface control remain necessary for improving ICE and full-cell stability.

The matching between pore structure and silicon deposition state determines cycling stability more directly than simply increasing silicon loading. Liu et al. [78] used CO_2_-derived carbon as the carbon source, regulated the pore structure of porous carbon hosts through KOH activation, and further conducted silane CVD and acetylene-derived outer carbon coating to prepare CVD-derived Si/C anode materials. This study showed that an appropriate micropore proportion is favorable for SiH_4_ adsorption, nanoscale silicon deposition, and spatial confinement by the carbon host. Mesopores are also needed because they support gas diffusion, electrolyte infiltration, and Li^+^ transport after silicon incorporation. When the mass ratio of carbon to KOH was 1:2, the Si/EC-1:2 material achieved a better balance among silicon loading, residual pore connectivity, and structural stability. It delivered a reversible specific capacity of 1448.7 mAh/g and retained 83.7% of its capacity after 300 cycles at 0.5 A/g. Increasing the activation ratio to 1:3 increased the silicon content but reduced the micropore proportion. The silicon distribution became less uniform, leading to capacity decay. This comparison shows that larger pore volume alone does not necessarily improve electrochemical performance. Excessive pore enlargement may weaken silicon confinement, reduce deposition uniformity, and lower electrode density. Thus, the host should retain suitable micropores for silicon nucleation, connected mesopores for transport, and reserved pore volume for expansion buffering.

Carbon coating further improves the stability of Si/C anodes, but its effectiveness also depends on the underlying carbon host structure. Wang et al. [79] constructed a mesocarbon microbead (MCMB)@Si@C sandwich structure. They used phenolic resin, polydopamine, and coating pitch as examples to compare the effects of different carbon sources on the electrode. In this structure, MCMB serves as a dense and mechanically stable carbon host, which is beneficial for electrode compaction and volumetric capacity. However, its limited internal pore volume provides less space for silicon expansion than highly porous hosts. Therefore, the outer carbon layer becomes important for compensating for insufficient pore buffering by improving interfacial protection and mechanical restraint. Different carbon sources produced carbon layers with different continuity, compactness, and ion-transport characteristics. Coating-pitch-derived carbon formed a continuous and dense protective layer. This carbon layer reduced direct contact between the electrolyte and the surface, improving the cycling performance of the battery. The pitch-derived amorphous carbon layer showed moderate flexibility and may help buffer the volume changes of silicon during lithiation and delithiation. The anode retained a reversible specific capacity of 352 mAh/g after 100 cycles at 0.2 A/g. This example indicates that dense carbon hosts are more favorable for electrode density and volumetric capacity, but their limited pore volume requires stronger coating protection. Excessive coating should still be avoided because it may reduce silicon content and increase Li^+^ transport resistance.

To gain deeper mechanistic insight into host-controlled silicon deposition and stress evolution, DFT calculations and finite element simulation have been used to examine how the surface structure and mechanical response of the carbon host affect electrochemical performance. Lv et al. [80] used DFT calculations and finite element simulation to clarify how a CNT-functionalized porous hard carbon (PCCN) host regulates CVD Si deposition and the subsequent electrochemical response. For comparison, porous hard carbon (PC) without CNT modification and nonporous hard carbon (C) were also prepared, leading to PCCN@SC, PC@SC, and C@SC composite anodes, where “SC” refers to the Si/C structure obtained after CVD Si deposition and subsequent outer carbon coating. During Si deposition, the adsorption energies of SiH_4_ on PCCN, PC, and C were −0.17, −0.19, and −0.33 eV, respectively, suggesting that defect- and oxygen-rich carbon surfaces may induce stronger localized silane adsorption. This strong adsorption may favor preferential SiH_4_ decomposition at specific sites and promote continuous Si particle growth. In contrast, the ordered lattice structure of CNTs in PCCN weakens such localized adsorption, allowing SiH_4_ to diffuse and nucleate more uniformly on the carbon host and thereby confining the Si particle size to 36–82 nm. From the viewpoint of host structure, PCCN combines pore volume for expansion buffering, connected channels for Li^+^ transport, and CNT-reinforced conductive pathways for electron transfer. Finite element simulation further suggests a more homogeneous von Mises stress distribution in PCCN@SC during lithiation. Dense C@SC lacks internal void space and is more prone to stress concentration, while PC@SC can release part of the expansion strain through pores but may still show local deformation around pore edges. In PCCN@SC, the CNT network may reinforce the mechanical rigidity of the porous host, reduce transport tortuosity, and cooperate with the pore structure to disperse Si-expansion-induced stress. As a result, Si size confinement, stress homogenization, and continuous conductive pathways may contribute to reduced interfacial fracture and particle pulverization while improving Li^+^ diffusion and charge-transfer kinetics, leading to lower charge-transfer resistance, higher Li^+^ diffusion coefficients, and more stable cycling performance.

**Figure 5:**
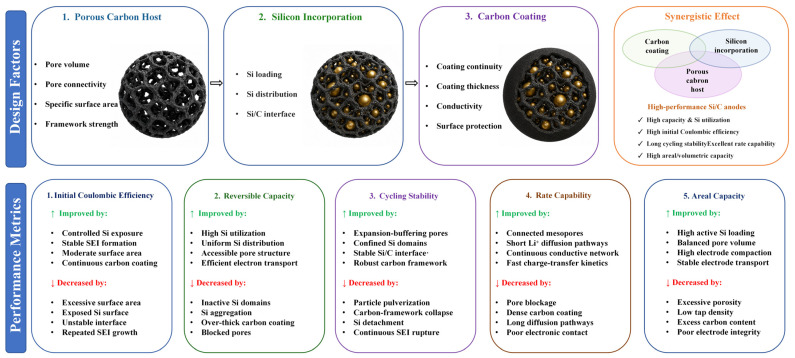
Structure–Performance Relationships in Si/C Anodes.

Overall, the general design of Si/C anodes should start from the target cell requirement, including reversible capacity, ICE, electrode density, areal capacity, rate capability, and allowable thickness expansion, as shown in Figure 5. Based on the target silicon content, the porous carbon host should reserve sufficient pore volume for silicon loading and lithiation-induced expansion, while retaining connected mesopores for electrolyte infiltration and Li^+^ transport. Micropores or small mesopores should be used to promote silicon precursor adsorption, nanoscale silicon deposition, and local confinement, but excessive surface area should be avoided because it increases irreversible SEI formation and lowers ICE. Macropores or larger voids can relieve electrode-level stress, but excessive large pores may reduce tap density, weaken silicon confinement, and decrease volumetric capacity. Therefore, pore size, pore volume, pore connectivity, surface area, tortuosity, and framework strength should be coordinated rather than independently maximized. Silicon incorporation should match the host structure: CVD is suitable for uniform deposition in connected pores, in situ formation is suitable for integrated Si/C interfaces, and mechanical compositing is suitable for low-cost scalable precursor organization. A subsequent carbon coating should further isolate silicon from the electrolyte, stabilize the SEI, maintain electronic pathways, and suppress electrode swelling, while avoiding overly thick coatings that reduce silicon content and hinder Li^+^ transport. An effective Si/C anode should balance silicon utilization, ion/electron transport, interfacial stability, volume buffering, electrode compaction, and scalable processing to achieve high capacity, high ICE, low expansion, and long cycling stability in both half cells and full cells.

**Figure 6:**
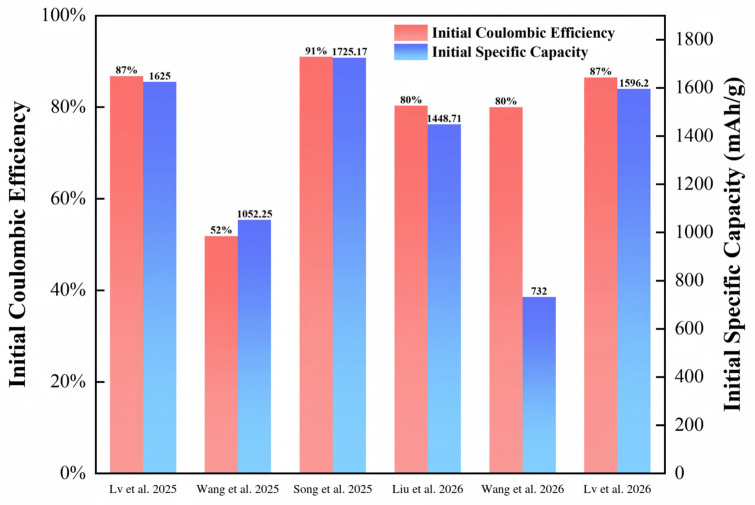
Comparison of initial Coulombic efficiency and initial specific capacity of representative Si/C anodes. The data were compiled from Refs. [74,75,77,78,79,80].

To more intuitively compare the differences among different structural designs in practical application metrics, Table 3 summarizes the silicon content, initial Coulombic efficiency, half-cell cycling performance, full-cell performance, and thickness expansion of representative Si/C anodes. To further facilitate a direct comparison of battery performance across different studies, Figure 6 compares the initial Coulombic efficiency and initial specific capacity reported for representative Si/C anodes. It should be noted that the C-rates listed in Table 3 follow the definitions reported in the original studies; because the actual current density corresponding to 1 C may vary among different works depending on the reference capacity, active-material loading, electrode configuration, and testing protocol, these C-rate values should be interpreted together with the specific experimental conditions when available. Structural designs that maintain balanced performance across multiple metrics are more suitable for evaluating the practical application value of Si/C anodes than designs that show high specific capacity only under simplified half-cell conditions.

## 5. Industrial Application and Challenges

The industrial value of porous-carbon-based Si/C anodes cannot be judged from coin-cell performance alone. It should be examined under high-loading electrodes, large-capacity pouch cells, and continuous manufacturing conditions. At the material level, porous carbon hosts regulate silicon dispersion, volume buffering, electron transport, and interfacial stability. At the electrode level, these structural functions need to be translated into high areal capacity, limited thickness expansion, suitable compaction density, and stable ion transport in thick electrodes. At the cell level, they must further support long cycle life, low swelling, and high gravimetric and volumetric energy density. Therefore, industrial application requires a transition from powder-structure optimization to electrode- and cell-level validation.

**Figure 7:**
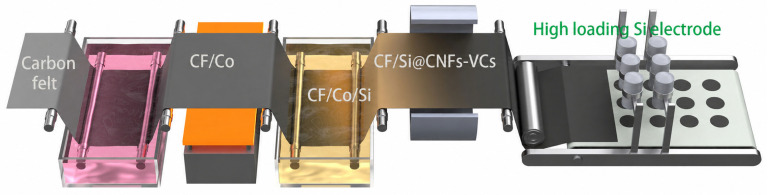
Schematic of the design and fabrication of a 3D CF/Si@CNFs–VCs composite fiber membrane. Reproduced from Ref. [81] under the terms of the Creative Commons Attribution 4.0 International License (CC BY 4.0).

Mu et al. [81] provided an example of this transition by constructing a high-areal-capacity Si/C anode using porous carbon felt as a three-dimensional supporting host, as shown in Figure 7. In this structure, Si nanoparticles were anchored in the carbon felt by vacuum filtration, and carbon nanofibers and vertical carbon nanosheets were further grown by thermal CVD. The carbon felt provided a macroporous framework for silicon loading and electrolyte penetration, while the carbon nanofibers and vertical carbon nanosheets formed interconnected conductive pathways. This multilevel host design reduced the transport limitations that commonly appear in thick electrodes and helped buffer silicon volume change at the electrode scale. The electrode achieved a high mass loading of over 30 mg/cm^2^, an ultrahigh areal capacity of 45.8 mAh/cm^2^, and retained 8.13 mAh/cm^2^ after 500 cycles. More importantly, the material was further validated in Ah-level NCM811//CF/Si@CNFs–VCs pouch cells, which showed an initial capacity of approximately 1.15 Ah and retained 79.34% of the capacity after 500 cycles at 1 C. The pouch cell exhibited only about 2% thickness variation after 500 cycles, indicating that the multi-level carbon network effectively suppressed Si-anode expansion at the device level, which is beneficial for maintaining volumetric energy density and long-term cycling stability.

Public information from industry also indicates that silicon confinement by porous carbon hosts has entered the stage of commercial validation. Group14’s SCC55^®^ (Group14 Si/C anode material) Si/C anode material uses a porous hard-carbon host to encapsulate amorphous nanosilicon. The internal pores of the particles buffer silicon volume changes during cycling and reduce side reactions and lithium loss caused by silicon exposure to the electrolyte [82,83,84]. At the electrode-design level, Sionic/Group14 further emphasized that high-energy Si-dominant cells require cathode loadings around 5 mAh/cm^2^ with uniform electrode thickness and roll-to-roll processing consistency. Sionic Energy has used SCC55^®^ as a 100% anode material and carried out validation in 20 Ah pouch cells. Cell-level swelling below 4% has been achieved, with reported engineering metrics of up to 370 Wh/kg, a volumetric energy density of 1000 Wh/L, and a cycle life of 600–1400 cycles [85]. In terms of material scale-up, Group14’s SCC55^®^ factory in Sangju, South Korea, has a designed annual production capacity of 2000 tons, corresponding to approximately 10 GWh of battery-material output [82]. These advances indicate that porous-carbon-host-regulated Si/C anodes have progressed from material development to electrode-level areal-loading optimization, cell-level validation and scaled production-capacity construction.

Industrial production of Si/C anodes has also been advanced by several Chinese companies. A published patent from Lanxi Zhide proposed a strategy in which silicon nanoparticles are loaded onto porous materials and then coated with carbon [86]. Published patents from Griffin and Guangdong Dowstone Silicon-Carbon Materials Technology Co., Ltd. show that their Si/C anode materials consist of porous carbon, nanosilicon inside the pores, and a surface carbon layer [87].

The large-scale industrial production of Si/C anodes has made considerable progress. Future development should further focus on large-format Ah-level pouch cells, supporting lithium-ion batteries with higher energy density and longer cycle life.

## 6. Conclusions and Perspectives

The electrochemical performance of Si/C composite anodes is governed by silicon incorporation, carbon host architecture, and interfacial structure. The preparation of porous carbon hosts has moved beyond simple pore formation and has gradually evolved into a structural design system that integrates silicon loading, volume buffering, and transport regulation. Hard-templating, soft-templating, combined hard- and soft-templating, template-free, and etching methods show distinct advantages in pore-structure replication, mesopore construction, hierarchical-pore coordination, endogenous pore formation, and pore refinement, respectively. These methods regulate the pore-size distribution, pore volume, pore connectivity, and structural stability of carbon hosts, with direct effects on silicon loading behavior, volume-buffering capability, ion transport, and interfacial reactions. The function of porous carbon should not be understood merely as increasing specific surface area. Instead, it needs to be matched with silicon deposition space, expansion-buffering capability, conductive continuity, and interfacial stability.

Silicon incorporation and carbon coating are critical to the long-term stability of porous-carbon-host-based Si/C anodes. CVD is well suited for regulating silicon deposition location, particle size, and interfacial contact, while in situ generation enables the synchronous formation of silicon phases and carbon-phase compositing. Mechanical compositing, by contrast, offers a low-cost and readily scalable route for precursor organization. After silicon incorporation, carbon coating can further enhance interfacial stability and structural integrity. Since different carbon sources produce coating layers with distinct functions, the coating design should not rely simply on increasing coating amount. Greater attention should be paid to matching the coating structure with specific functional requirements, including electrolyte isolation, electron transport, stress accommodation, and interface stabilization.

The development of Si/C anodes should move beyond evaluation based solely on high specific capacity in laboratory half cells and toward comprehensive validation under scalable preparation and practical electrode conditions. From an application perspective, maintaining reversible specific capacity, initial Coulombic efficiency, and cycling stability at relatively high silicon content, high compaction density, and stable electrode-processing conditions is more meaningful than achieving an isolated half-cell capacity value. Batch consistency, scale-up stability, and electrode-processing compatibility should receive greater attention, as these factors directly determine performance reproducibility during large-scale preparation. In parallel, cycle life, electrode thickness expansion, impedance growth, and capacity retention in Ah-level cells should be incorporated as key evaluation metrics. Establishing a reliable correlation among material structural design, preparation process, and cell-level performance will be essential for moving Si/C anodes from laboratory optimization toward practical application.

## Figures and Tables

**Table 1:** Comparison of preparation methods for porous carbon hosts.

Method	Advantages	Limitations	Structural Targets
Hard-Templating Method	High pore-structure accuracy, designable pore size, and regular pore morphology	Multiple processing steps, high template-removal cost, and relatively heavy environmental burden	Regular mesopores, connected networks, and directionally designed hierarchical pores
Soft-Templating Method	Flexible mesopore regulation, simultaneous surface-chemistry design, and relatively simplified processing	Sensitive assembly conditions, insufficient ordering, and weak batch stability	Mesoporous networks, hollow structures, and surface-functionalized structures
Combined Hard- and Soft-Templating Method	High structural design freedom, partitioned regulation of external hosts and internal pores, and cooperative construction of multiscale pores	Complex parameter coupling, low reproducibility, and high difficulty in scale-up preparation	Cavity structures, mesoporous shells, hierarchical pore networks, and composite hosts
Template-Free Method	Short processing chain, low cost, good scale-up potential, and simultaneous pore formation and host generation	Weak pore-structure predictability, strong precursor dependence, and insufficient batch consistency	Endogenous mesopores, inherited natural host structures, and continuous porous networks
Etching Method	Active pore-structure regulation, strong continuous pore-expansion capability, and high ability for hierarchical-pore refinement	Sensitive reaction conditions, high requirements for process control, and risk of excessive etching	Pore-rich carbon hosts, continuously expanded pore structures, and refined hierarchical pores

**Table 2:** Comparison of representative silicon introduction strategies in Si/C composite anodes.

Method	Advantages	Limitations	Structural Targets
Chemical Vapor Deposition Method	Controllable deposition location, tunable particle size, close interfacial bonding, and high structural uniformity	High equipment cost, narrow processing window, strong parameter sensitivity, and difficult continuous-process control	Directed interfacial construction, confined deposition, and highly uniform composite structures
In Situ Generation Method	Synchronous phase formation and compositing, uniform composition distribution, good interfacial continuity, and high structural integration	Strong precursor dependence, narrow reaction window, complex process coupling, and insufficient scale-up stability	In situ phase formation, continuous interface, and synchronous carbon-layer construction
Mechanical Compositing Method	Simple process, strong raw-material adaptability, low cost, and good scale-up potential	Dominant physical contact, limited interfacial coupling, easy agglomeration, and possible defect and impurity introduction	Basic compositing, precursor homogenization, and scalable preparation

**Table 3:** Comprehensive application performance comparison of silicon/carbon anodes in lithium-ion batteries.

Preparation Method	Si Content	Initial Coulombic Efficiency	Half-Cell Cycling Performance	Full-Cell Cycling Performance	Thickness Expansion
CVD silicon incorporation + pitch-derived carbon coating [74]	≈57.1 wt%	86.8%	Initial specific capacity of 1625 mAh/g, 680 mAh/g after 70 cycles at 0.3 C, with a capacity retention of 57.0%	Capacity retention of about 55–56% after 100 cycles	62.7%, from 10.2 to 16.6 μm after 70 cycles
CVD silicon incorporation + rGO self-assembled coating [75]	≈71.7 wt%	51.85%	1052.25 mAh/g after 140 cycles at 0.2 A/g, with a retention of 90.44%. 698.25 mAh/g after 900 cycles at 1 A/g, with a retention of 78.2%	Not reported	About 7.4%, from 21.65 to 23.25 μm after 300 cycles
CVD silicon incorporation + methane CVD carbon coating [77]	≈64.9 wt%	91.02%	Initial discharge specific capacity of 1725.17 mAh/g, 898.33 mAh/g after 100 cycles at 0.5 C, with a retention of 75.66%	152.81 mAh/g after 150 cycles at 1 C, with a capacity retention of 62.25%	35.86%, from 19.55 to 26.56 μm after 100 cycles
CVD silicon incorporation + acetylene CVD carbon coating [78]	≈47.1 wt%	80.32%	Specific capacity of 1448.71 mAh/g at 0.5 A/g, 1201.5 mAh/g after 300 cycles at 0.5 A/g, with a retention of 83.7%	Capacity retention of 56% after 200 cycles at 0.25 C	31.5% after 100 cycles
Carbon coating using different carbon sources [79]	<15 wt%	80%	Initial discharge specific capacity of 732 mAh/g, 352 mAh/g after 100 cycles at 0.2 A/g, with a retention of 48.1%	Not reported	Not reported
CVD silicon incorporation + methane CVD carbon coating on CNT-functionalized porous hard carbon host [80]	≈55.3 wt%	86.5%	Initial discharge specific capacity of 1596.2 mAh/g, 1229.7 mAh/g after activation for three cycles, with a capacity retention of 85.4% after 100 cycles at 1 A/g; capacity retention of 84.9% after 500 cycles at 2 A/g	Initial discharge capacity of 147.3 mAh/g, with a capacity retention of 86.9% after 110 cycles at 0.5 C	37.3%, from 12.6 to 17.3 μm after 100 cycles

## Data Availability

No new data were created or analyzed in this study. Data sharing is not applicable to this article.
